# Supplementary material for: Epidermal necrolysis (Stevens-Johnson syndrome/ toxic epidermal necrolysis) as extensive boundary violation: A qualitative study on the illness experience and care needs of survivors in the context of the first German guideline
Source: PLoS One. 2025 Oct 14;20(10):e0333636. doi: 10.1371/journal.pone.0333636 (PMC12520350; doi:10.1371/journal.pone.0333636)
Supplement: S1 File — (DOCX) [file pone.0333636.s001.docx]

**Additional information regarding recording and software used**

Interviews were audio-taped for in-person meetings or recorded through the video conferencing software Skype for Business in the case of online sessions. Subsequently, recordings were transcribed verbatim by the interviewer, who also coded and analyzed the transcripts. To organise the transcripts, simplify the comparison of coded segments, and perform queries on the data, we used the software package MAXQDA 2022. The software also provided a segment-by-segment synchronization between the recorded audio and the transcript, which allowed us to selectively consider the richer tonal nuances of the original recording rather than relying solely on the written transcripts

**Table S1: Sensitizing concepts from the initial focus group**

| Initial contact / diagnosis | Acute phase | Post-acute / follow-up care | Impact on quality of life / psychological impact | General information flow |
| --- | --- | --- | --- | --- |
| - Delayed diagnosis and transfer to ICU / burn unit - Incomplete communication of necessary treatment steps - Lack of early involvement of an ophthalmologist | - Deficits in physician-patient communication (inadequate language, jargon) - Dampened state of consciousness in the first weeks makes it difficult to process information - Disturbed sleep; restrictive supply of sleep medication - Pronounced feeling of shame (bandages as only coverage) - Pain relief of central importance; no clear understanding why certain measures are being taken - Inadequate/insufficient information on prognosis (e.g., presumed hospital discharge) | - Limited care provided for ocular conditions in follow-up rehabilitation - Time of follow-up rehabilitation not always optimal, spatial distance to relatives perceived as stressful - No measures against cognitive impact of disease provided (e.g., concentration / memory training) | - Psychological distress due to pain-related trauma - Lack of psychological support for family members (sometimes more emotionally affected than patients due to clearer perception of critical phase) - Lack of understanding of the exceptional character of the disease in psychotherapists (e.g., “just an allergy”) - Fear of taking medications even in infection-induced cases - Difficulties in communicating the impact of the disease on one’s life; feeling of social alienation | - Information overwhelming / not individualized / problem-oriented, inappropriately complex - Increased need for safety regarding newly prescribed medication - Difficulties in finding EN-experts (post-acute care) - Desire for regular “check-up visits” - Patients do not feel “seen” during phone consultations - Need/wish for exchange with other EN patients |

**Table S2: Additional interview data demonstrating themes related to stress limits/breaking points**

| **Feeling overwhelmed**  *DZH001 (family member): Respect to her - still, really - but she often said, ‘Why don't they put me in a coma? I want to go into a coma, I want to go into a coma?’ [I] don’t understand that, either. And I actually asked the question once, but it wasn’t answered.*  **Loss of agency**  *DZH003a (patient): I also think it's important that you realise - I mean, the whole time I was in the hospital, I could never really change anything or determine how things were. And then to realise, ok, I play sports, I can decide my own fate, about my body, uh, to really realise that and then to have power over it, I think that's worth its weight in gold - well, that's really valuable.* |
| --- |

**Table S3: Additional interview data demonstrating themes related to the boundary between fantasy and reality**

| **Dream-like experience**  *DZH001a (survivor): So, with respect to these dreams, when I've slept and dreamed it was like… My dreams - they always took place in the outside world - and then I still had my arms and then I was running around everywhere (laughs). But then I woke up again, and I was back in the hospital. So, reality, was always just there in the dream...*  **Normality**  *DZH003a: And then, at the time when I was already feeling better, I went out and... There was a cafeteria where you could eat something cool (laughs). That was also pretty relaxed, and just something normal to experience at a time when everything is so... not normal."* |
| --- |

**Table S4: Additional interview data demonstrating themes related to limits of understanding**

| **Reduced situational understanding**  *DZH001a: Yes, so, if everyone tells you something different. For example, a dermatologist who tells me it will go away after three days, then the next one comes along and says I have to take more cortisone. The other doctor says, yes, maybe it's the cortisone causing it... So, somehow nobody has any idea what's going on. No one can tell you what you have. And that's just exhausting... One time you're relieved, and then it’s not happening after all. Your hope is always destroyed that it will... Very exhausting in any case.*  *---*  *DZH006: Well, I thought that the skin around the penis was quickly affected... well, that it was quickly inflamed. And this is also something where I have to say afterward, now that it has now healed up so scarred... that we now have to decide again whether to have surgery or no surgery. Yes, it's just these, these aftereffects, these consequences... that you don't know anything about in advance, or... you don't know what's going to happen - yes, now we're a year later - what can still come in terms of consequences.*  *RH: Would you have liked to have known that beforehand, or...?*  *DZH006: I think I would have liked to have known in advance what might happen so that I could have reacted more quickly...*  **Existential distress**  *RH: How did he himself feel about it, apart from the physical aspect? How did he feel about it?*  *DZH006 (family member): He thought it was really bad... and then he asked why I had to... get something like that? And, um... Yes, he really put up with all of it. When people asked him how he was, he always said he was fine.* |
| --- |

**Table S5: Additional interview data demonstrating themes related to social boundaries**

| **Self-determination**  *RH (interviewer): "Is there anything where you would say: that would have helped me… because... I don't have to handle it all by myself, or something?"*  *DZH005a (family member): "Probably not.... probably I wouldn't have let that happen either. I actually wanted to... I wanted to be there for her, I wanted to (unintelligible). Even if I now, um... if I was partly totally exhausted, and I.... um, for example, I've had terrible back pain since then..."*  *RH: "But you still didn't want to hand it over to someone else?"*  *DZH005a: "Nah. I wouldn't do that either. So... if I could do it again, I also wouldn't give it up."* |
| --- |

**Table S6: Additional interview data demonstrating the theme of temporal boundaries**

| **Patience**  *DZH006 (survivor): What was important to him is that you need a lot of time for everything. So, the nurses there had to have a lot of patience (laughter). He demanded that again and again. That was actually... He was like 'Go slowly. I... can you please wait again'. So, this... um, slow everything down.* |
| --- |
