# Supplementary material for: Epidermal necrolysis (Stevens-Johnson syndrome/ toxic epidermal necrolysis) as extensive boundary violation: A qualitative study on the illness experience and care needs of survivors in the context of the first German guideline
Source: PLoS One. 2025 Oct 14;20(10):e0333636. doi: 10.1371/journal.pone.0333636 (PMC12520350; doi:10.1371/journal.pone.0333636)
Supplement: Attachment — Submitted filename: Response to Reviewers.docx [file pone.0333636.s003.docx]

DIVISION OF EVIDENCE BASED MEDICINE

IN DERMATOLOGY (DEBM)

**KLINIK FÜR DERMATOLOGIE,**

**VENEROLOGIE UND ALLERGOLOGIE**

|  | Klinik mit zertifiziertem Qualitätsmanagementsystem nach  DIN EN ISO 9001:2008 |
| --- | --- |

Prof. Dr. med. A. Nast

alexander.nast@charite.de

Tel.:	+49 30 450 618 313

Fax:	+49 30 450 518 977

www.derma.charite.de

www.debm.charite.de

Berlin, 23.08.25

Dear Dr. Hosohata,

enclosed please find the revised manuscript of our study

**‘Epidermal Necrolysis (Stevens-Johnson Syndrome / Toxic Epidermal Necrolysis) as Extensive Boundary Violation: A Qualitative Study on the Illness Experience and Care Needs of Survivors in the Context of the First German Guideline’**

by the authors Heuer, R., Paulmann, M., Mockenhaupt, M., Nast, A. All authors have read and approved the revised manuscript, its content, and its submission to *PLOS ONE*. We would like to submit the revised article to *PLOS ONE* for publication as an original research article.

We have carefully considered the valuable feedback provided by the reviewers. Wherever possible, we have incorporated their suggestions to improve the manuscript. You will find a detailed outline of our revisions below.

Thank you for your time and consideration. We look forward to your feedback on our revised manuscript.

With kind regards,

Ruben Heuer

Maren Paulmann

Maja Mockenhaupt

Alexander Nast

**Revised funding statement (RH, MP, MM, AN):**

This research was funded through the federal joint committee of the joint self-government of physicians, dentists, hospitals, and health insurance funds in Germany (<https://www.g-ba.de/>; grant number 01VSF21002). The funders had no role in study design, data collection and analysis, decision to publish, or preparation of the manuscript.

**Revised competing interests statement (RH, MP, MM, AN):**

Ruben Heuer, Maren Paulmann, and Alexander Nast declare no potential conflicts of interest involving the work under consideration for publication.

Maja Mockenhaupt reported serving on a scientific advisory board for Boehringer Ingelheim and Merck AG; the German Registry on severe skin reactions (dZh) received financial support for research (through contracts with the University of Freiburg) from several pharmaceutical companies for the provision of safety information related to drugs manufactured or distributed by them.

The funder provided support in the form of salaries but did not have any additional role in the study design, data collection and analysis, decision to publish, or preparation of the manuscript. The specific roles of these authors are articulated in the ‘author contributions’ section.

This does not alter our adherence to PLOS ONE policies on sharing data and materials.

**Response to reviewers:**

Reviewer #1: I like the study you presented, I believe there is an important role to deepening the understanding of the social, personal and emotional impact of SJS/TEN in our patients (also applicable to other diagnoses). However, the manuscript is dense to read and I believe that the information can be delivered in a shorter and less redundant manuscript. I like the figure you provided and that should be added in the paper and could help summarize the words from the paragraphs. Make the paper lighter to read and more engaging because the information is valuable and should be available to the medical community.

Reply:

Thank you very much for your detailed and thoughtful feedback. As you suggested, we moved Supplementary Figure 1 to the main manuscript. Additionally, we have split up long sentences, removed redundancies, and worked to improve overall readability. In addressing these points, as well as incorporating the suggestions from Reviewer #2, we were unfortunately unable to reduce the overall word count further without compromising the clarity or completeness of the content. We hope, however, that the revisions have enhanced the flow and accessibility of the manuscript, and that its current length is justified by the relevance of the material presented.

Reviewer #2:

General comments

The topic is interesting, and enlightening, and under-recognized in literature. The gained insights would help in better managing patients with epidermal necrolysis. The approach to the topic is sound and the procedure followed is thorough and accurate. Data presentation is comprehensive and logical. The language used is appropriate and correct.

Some questions need to be addressed in general. These include:

1) How does experience of EN differ from that of skin burns?

2) Why weren`t there comparisons with previous literature on the topic in the discussion section? with emphasis on what knowledge gaps addressed in this research that was lacking in other similar studies.

3) Exclusion criteria were not mentioned in the manuscript.

4) Sex of the participants also is not mentioned, and whether there were differences in disease perception and outcomes discussed between males and females?

5) The impact of age category also on the discussed variables is not discussed.

6) I think that a separation of the perspective of caregivers from that of the patients should have been done, rather than collectively presenting their data.

7) Effects of oral mucosal affection on the patients that led to feeding restrictions, necessity for tube feeding sometimes, are not addressed by the manuscript.

Specific comments

Introduction

8) P2, line 3: “alongside pre-reaction or even improved levels” please explain, and write in a clearer way.

Reply:

Thank you for your detailed and valuable feedback. We have addressed your suggestions as follows:

1) We have fleshed out the introductory passage on EN as such:

“In contrast to burn injuries, EN presents as a progressive disease that can leave patients overwhelmed with rapid deterioration [1–3]. Different from burn survivors, many EN-patients also experience extensive mucosal damage, leading to complications of the oral cavity, eyes, and genitals. Among those, conjunctival or corneal epithelial sloughing requiring rigorous ocular surface protection measures, and painful oral erosions impairing oral nutrition are some of the most debilitating [1–3]. Long-term sequelae that demand continued specialist care are also common, including ocular surface scarring, vulvovaginal pain or phimosis [4–8]. Moreover, survivors can suffer deeper psychological consequences of EN, as anxiety around recurrence and avoidance of potentially causative medications can create tremendous emotional strain [4,6–13].”

2) We have added the following passage in the discussion section:

“In contrast to earlier studies that reported attitudes of mistrust in the medical system associated with the predominantly iatrogenic nature of the disease, [14,15] our results paint a more nuanced picture. Even though many of the participants experienced feelings of powerlessness and dissatisfaction with some of the treatment decisions made, narratives of empowerment and a willingness to navigate the ongoing recovery collaboratively with medical providers were equally prominent. One possible explanation is that most of our participants had received early guidance through the German Registry of Severe Skin Reactions, including extensive advice on safe medical treatment and reassurance about the statistically very low risk of recurrence. For many, the registry remained a consistent point of contact, offering ongoing support as new medical challenges arose.”

3) We have added the following sentences to the methods section:

“We included survivors aged 16 and over with a confirmed diagnosis of EN, and, where applicable, their relatives. We excluded individuals with significant cognitive impairments that could compromise their capacity to provide informed consent.”

4 and 5) We have added the following paragraph to the discussion section:

“Limitations also apply to the investigation of gender- and age-related differences in the illness experience, which was not undertaken in our study. Even though participants’ reports touched on matters of social identity and certain medical categories, such as reproductive health, which could have been read in a gender- or age-sensitive way, we decided not to conduct such an analysis. Primarily, our decision rests on the wish to avoid stereotyping based on a small sample, for example interpreting the differential expression of recovery goals between participants (i.e., recovery of skin appearance vs physical fitness) as necessarily attributable to gender or age. However, our dataset contains indications of gender- and age-specific psychosocial impacts, even if these were only addressed implicitly. Such issues could be explored more systematically in future qualitative and quantitative research. It is worth noting that our sample included a relatively balanced distribution of female (n = 8) and male (n = 6) participants in different age groups (<25: n = 3, 25-59: n = 8, >60: n = 2) reducing the likelihood of underrepresentation. We hope that future studies building on these preliminary findings can provide a more comprehensive and accurate picture.”

Additionally, we have adapted Table 1 to include the participants’ sex as well as to allow for cross-referencing their quotes with the respective demographic information:

*Table 1: Recovery periods at the time of interview, sex, and age of participants (green=female, blue=male; bold=survivor, plain type=family member):*

|  | Age: <25 | Age: 25-59 | Age: ≥60 |
| --- | --- | --- | --- |
| ≤2 years after reaction | **DZH001a**  **DZH002** | **DZH005 KFA002** KFA002a  DZH001  DZH005a  DZH006 | **CCM001**  **DZH004** |
| 3-10 years after reaction | **DZH003a** | **DZH007**  DZH003 | **-** |
| >10 years after reaction | **-** | **KFA001** | **-** |

6) We appreciate this suggestion. However, the fact that caregivers’ and survivors’ contributions are not treated separately reflects a deliberate decision on our part. There are two main reasons for this. First, we sampled multiple survivors without their caregivers and vice versa. Analysing the data separately would run the risk of juxtaposing experiences that may appear related but are, in fact, independent. Second, the EN-accounts in our study mostly depict a shared illness experience across both groups, with many describing strengthened familial bonds. Where notable differences emerged, for example in the differing timing of feelings of overwhelm between survivors and caregivers in the section *Stress Limits/Breaking Points*, we highlighted these distinctions explicitly in the text.

7) Except for one participant, who reported persistent sensitivities to acidic foods, none of our interview partners referred to nutritional restrictions during the time of hospitalisation or after. This may either point to a biased sample regarding this particular complication of EN or a feature of the recollection of the early illness experience (e.g., incomplete memory due to sedation, suppression of traumatic or precedence of more impactful memories). We have expanded the introductory section to describe different mucosal manifestations and referenced the requirement of enteral feeding in some patients. We now also briefly discuss the issue food sensitivities in the section *Stress Limits/Breaking Points*.

8) We have changed the respective sentence to read as follows:

“Accordingly, studies report long-term reductions in quality of life in EN survivors while some maintain or even exceed their self-reported levels of psychosocial wellbeing [4,9–13].”

**References**

1.	Mockenhaupt M. Bullous Drug Reactions. Acta Derm Venereol. 2020;100: 122–134. doi:10.2340/00015555-3408

2.	Creamer D, Walsh SA, Dziewulski P, Exton LS, Lee HY, Dart JKG, et al. UK guidelines for the management of Stevens-Johnson syndrome/toxic epidermal necrolysis in adults 2016. J Plast Reconstr Aesthetic Surg JPRAS. 2016;69: e119–e153. doi:10.1016/j.bjps.2016.01.034

3.	Lerch M, Mainetti C, Terziroli Beretta-Piccoli B, Harr T. Current Perspectives on Stevens-Johnson Syndrome and Toxic Epidermal Necrolysis. Clin Rev Allergy Immunol. 2018;54: 147–176. doi:10.1007/s12016-017-8654-z

4.	Hoffman M, Chansky PB, Bashyam AR, Boettler MA, Challa N, Dominguez A, et al. Long-term Physical and Psychological Outcomes of Stevens-Johnson Syndrome/Toxic Epidermal Necrolysis. JAMA Dermatol. 2021;157: 712. doi:10.1001/jamadermatol.2021.1136

5.	Olteanu C, Shear NH, Chew HF, Hashimoto R, Alhusayen R, Whyte-Croasdaile S, et al. Severe Physical Complications among Survivors of Stevens–Johnson Syndrome and Toxic Epidermal Necrolysis. Drug Saf. 2018;41: 277–284. doi:10.1007/s40264-017-0608-0

6.	Lee HY, Walsh SA, Creamer D. Long-term complications of Stevens-Johnson syndrome/toxic epidermal necrolysis (SJS/TEN): the spectrum of chronic problems in patients who survive an episode of SJS/TEN necessitates multidisciplinary follow-up. Br J Dermatol. 2017;177: 924–935. doi:10.1111/bjd.15360

7.	Ingen‐Housz‐Oro S, Alves A, Colin A, Ouedraogo R, Layese R, Canoui‐Poitrine F, et al. Health‐related quality of life and long‐term sequelae in survivors of epidermal necrolysis: an observational study of 57 patients. Br J Dermatol. 2020;182: 916–926. doi:10.1111/bjd.18387

8.	Lefaucheur J ‐P., Valeyrie‐Allanore L, Ng Wing Tin S, Abgrall G, Colin A, Hajj C, et al. Chronic pain: a long‐term sequela of epidermal necrolysis (Stevens–Johnson syndrome/toxic epidermal necrolysis) – prevalence, clinical characteristics and risk factors. J Eur Acad Dermatol Venereol. 2021;35: 188–194.

9.	Chiang C, Hung C, Chen Y, Chen C, Soong C, Liu S, et al. Stevens–Johnson syndrome and toxic epidermal necrolysis associated with increased risk of developing psychiatric disorders: A retrospective population‐based cohort study in Taiwan. J Dermatol. 2022;49: 979–987. doi:10.1111/1346-8138.16487

10.	Zitha E. Prevalence of anxiety and depression in a predominantly HIV-infected population with severe cutaneous adverse drug reactions. Master Thesis, University of Cape Town. 2016.

11.	Hefez L, Zaghbib K, Sbidian E, Valeyrie‐Allanore L, Allain M, Duong TA, et al. Post‐traumatic stress disorder in Stevens–Johnson syndrome and toxic epidermal necrolysis: prevalence and risk factors. A prospective study of 31 patients. Br J Dermatol. 2019;180: 1206–1213. doi:10.1111/bjd.17267

12.	Dodiuk-Gad RP, Olteanu C, Feinstein A, Hashimoto R, Alhusayen R, Whyte-Croasdaile S, et al. Major psychological complications and decreased health-related quality of life among survivors of Stevens-Johnson syndrome and toxic epidermal necrolysis. Br J Dermatol. 2016;175: 422–424. doi:10.1111/bjd.14799

13.	O’Reilly P, Kennedy C, Meskell P, Coffey A, Delaunois I, Dore L, et al. The psychological impact of Stevens–Johnson syndrome and toxic epidermal necrolysis on patients’ lives: a Critically Appraised Topic*. Br J Dermatol. 2020;183: 452–461. doi:10.1111/bjd.18746

14.	Butt TF, Cox AR, Lewis H, Ferner RE. Patient Experiences of Serious Adverse Drug Reactions and Their Attitudes to Medicines: A Qualitative Study of Survivors of Stevens-Johnson Syndrome and Toxic Epidermal Necrolysis in the UK. Drug Saf. 2011;34: 319–328. doi:10.2165/11588460-000000000-00000

15.	Butt TF, Cox AR, Oyebode JR, Ferner RE. Internet Accounts of Serious Adverse Drug Reactions: A Study of Experiences of Stevens-Johnson Syndrome and Toxic Epidermal Necrolysis. Drug Saf. 2012;35: 1159–1170. doi:10.1007/BF03262001
